# Ryanodine‐ and CaMKII‐dependent release of endogenous CGRP induces an increase in acetylcholine quantal size in neuromuscular junctions of mice

**DOI:** 10.1002/brb3.1058

**Published:** 2018-07-06

**Authors:** Alexander E. Gaydukov, Olga P. Balezina

**Affiliations:** ^1^ Department of Human and Animal Physiology Biological Faculty Lomonosov Moscow State University Moscow Russia; ^2^ Department of Physiology Russian National Research Medical University Moscow Russia

**Keywords:** Ca/Calmodulin‐dependent kinase II, calcitonin gene‐related peptide, neuromuscular junction, quantal size, ryanodine

## Abstract

**Objective:**

The aim of this study was to identify the mechanism responsible for an increase in miniature endplate potentials (MEPPs) amplitude, induced by ryanodine as an agonist of ryanodine receptors in mouse motor nerve terminals.

**Methods:**

Using intracellular microelectrode recordings of MEPPs and evoked endplate potentials (EPPs), the changes in spontaneous and evoked acetylcholine release in motor synapses of mouse diaphragm neuromuscular preparations were studied.

**Results:**

Ryanodine (0.1 μM) increased both the amplitudes of MEPPs and EPPs to a similar extent (up to 130% compared to control). The ryanodine effect was prevented by blockage of receptors of calcitonin gene‐related peptide (CGRP) by a truncated peptide CGRP_8‐37_. Endogenous CGRP is stored in large dense‐core vesicles in motor nerve terminals and may be released as a co‐transmitter. The ryanodine‐induced increase in MEPPs amplitude may be fully prevented by inhibition of vesicular acetylcholine transporter by vesamicol or by blocking the activity of protein kinase A with H‐89, suggesting that endogenous CGRP is released in response to the activation of ryanodine receptors. Activation of CGRP receptors can, in turn, upregulate the loading of acetylcholine into synaptic vesicles, which will increase the quantal size. This new feature of endogenous CGRP activity looks similar to recently described action of exogenous CGRP in motor synapses of mice. The ryanodine effect was prevented by inhibitors of Ca/Calmodulin‐dependent kinase II (CaMKII) KN‐62 or KN‐93. Inhibition of CaMKII did not prevent the increase in MEPPs amplitude, which was caused by exogenous CGRP.

**Conclusions:**

We propose that the activity of presynaptic CaMKII is necessary for the ryanodine‐stimulated release of endogenous CGRP from motor nerve terminals, but CaMKII does not participate in signaling downstream the activation of CGRP‐receptors followed by quantal size increase.

## 1 INTRODUCTION

Recently, we have found that ryanodine‐stimulated calcium release from intracellular calcium stores initiates a calcium‐dependent increase in MEPP amplitude and ACh quantal size in mouse neuromuscular junctions (NMJs) (Skiteva, Lapteva, & Balezina, 2012). Both the mechanism of this ryanodine effect and possible ryanodine influence on the multiquantal evoked ACh release remain unknown.

Currently, several substances are known to increase the MEPPs amplitude when exogenously applied to mammalian motor synapses: γ‐S‐ATP (Bogacheva & Balezina, 2015), TGF‐β2 (Fong et al., 2010), agonists of endocannabinoid receptors (Morsch et al., 2018), BDNF (Gaydukov, Akutin, Bogacheva, & Balezina, 2018), calcitonin gene‐related peptide (CGRP) (Gaydukov, Bogacheva, & Balezina, 2016). Of these, CGRP is of special interest because this 37‐amino acid neuropeptide has been found in large dense‐core vesicles (LDCVs) in mammalian motor nerve terminals (Csillik et al., 1993; Matteoli et al., 1988) and is reported to be released as a co‐transmitter in response to sustained depolarization or intense nerve stimulation (Sakaguchi, Inaishi, Kashihara, & Kuno, 1991; Sala, Andreose, Fumagalli, & Lømo, 1995; Uchida et al., 1990) providing the wide spectrum of acute and neurotrophic influences (Buffelli, Pasino, & Cangiano, 2001; Changeux, Duclert, & Sekine, 1992; Correia‐de‐Sá & Ribeiro, 1994; Fernandez, Ross, & Nadelhaft, 1999; Kimura, Okazaki, & Nojima, 1997; Machado et al., 2016; Rossi, Dickerson, & Rotundo, 2003; Salim, Dezaki, Tsuneki, Abdel‐Zaher, & Kimura, 1998). Possible release of endogenous CGRP in response to ryanodine application at resting motor synapses and acute physiological consequences of this release on quantal ACh secretion have not been described yet. The aim of this study was to test the hypothesis that ryanodine can trigger calcium‐dependent release of CGRP from motor nerve terminals of mice and further presynaptic action of endogenous peptide may underlie the increase in MEPP amplitude and ACh quantal size.

We found that the ryanodine‐induced increase in MEPP amplitude and ACh quantal size is associated with the release of endogenous CGRP. Using pharmacological assays, we also provide evidence of specific role of presynaptic CaMKII, whose activation and leads to exocytosis of endogenous CGRP from mouse NMJ. We also found for the first time that endogenous CGRP, which was released into synaptic cleft of motor synapses, may act presynaptically stimulating the loading of ACh into synaptic vesicles.

## 2 METHODS AND MATERIALS

### 2.1 Preparation and solutions

Experiments were performed with classical neuromuscular preparations (diaphragm muscle supplied with phrenic nerve) isolated from adult BALB/c mice of either sex (25–30 g) 5–6 weeks old. The animals were obtained from the Laboratory of experimental animals, Department of Biology, Moscow State University (Moscow, Russia), and were kept according with the European Communities Council Directive from 24 November 1986 (86/609/EEC). All experimental procedures were approved by the Bioethics Committee of The Department of Biology at Moscow State University. The mice were sacrificed by quick decapitation. Left hemidiaphragm with the terminal branches of phrenic nerve was excised, mounted to a 3‐ml recording chamber, and superfused with Liley solution (mM: NaCl—135, KCl—4, NaH_2_PO_4_—0.9, CaCl_2_—2, MgCl_2_—1, NaHCO_3_—16.3, glucose—11, pH—7.2–7.4, gassed with 95% O_2_/5% CO_2_) via bath perfusion system (0.5 ml/min). All experiments were performed at room temperature (20–22°C).

### 2.2 Electrophysiological recordings

Intracellular recordings of endplate potentials (MEPPs and EPPs) were performed using sharp glass microelectrodes filled with 2.5 M KCl. Tip resistance of microelectrodes was 20–25 MΩ. Appearance of MEPPs with rise time (10%–90%) <1 ms was indicative of the muscle fiber impalement near endplate region. We recorded MEPPs for 180 s from muscle fibers with resting membrane potential (RMP) more negative than −60 mV. RMP was continuously monitored throughout the MEPP recordings in each synapse. If the value of RMP was not stable and decreased by more than 5 mV, the recording was stopped and the data acquired from this synapse were not included into the sample population for further analysis. Bridge balance and microelectrode capacitance neutralization was performed throughout the entire experiment.

Experiments on ACh multiquantal synchronous release evoked by stimulation of phrenic nerve were performed with cut neuromuscular preparations to prevent contraction as well as to record both MEPPs and EPPs from the same synapse (Barstad & Lilleheil, 1968). After the dissection of the fibers, the preparation was washed thoroughly for at least 1 hr in a large volume of the Liley solution (>150 ml). This procedure prevented the blockage of the action potential conduction. RMP was stabilized at a lower level (<−50 mV) than in intact fibers. Cutting of the fibers was not performed in preparations when only spontaneous ACh release was recorded. The phrenic nerve was stimulated with suprathreshold stimuli at a frequency of 0.3 Hz and with duration of 0.08–0.1 ms using two silver electrodes connected with an STG4002 stimulator (Multichannel Systems GmbH, Reutlingen, Germany). At least 30 EPPs were recorded in each synapse studied, and MEPPs were recorded for 60 s immediately before the nerve stimulation. Mean value of the MEPP amplitudes recorded within this period was used for the calculation of the quantal content of the subsequently recorded EPPs.

MEPPs and EPPs were amplified with Axoclamp 900A (Molecular Devices, Sunnyvale, CA, USA) or Neuroprobe Amplifier Model 1600 (A‐M Systems, Sequim, WA, USA) and digitized using a Digidata 1440A interface with pCLAMP10 software (Molecular Devices, Sunnyvale, CA, USA, http://scicrunch.org/resolver/SCR_011323) or a E‐154 interface with PowerGraph 3.3 software (L‐Card, Moscow, Russia) and then stored and analyzed using MiniAnalysis software (Synaptosoft, Fort Lee, NJ, USA, http://scicrunch.org/resolver/SCR_002184).

In each experimental series, at least three neuromuscular preparations were used. MEPPs (or MEPPs and EPPs) from 5 or more different synapses were recorded as control. After that, the perfusion solution was changed to the drug‐containing test solutions and synaptic activity of various synapses was recorded for 0.5–1.5 hr.

### 2.3 Data analysis

We estimated RMP of muscle fibers, MEPP and EPP amplitude and time course, and the MEPP frequency. When only spontaneous ACh release was studied, the MEPP amplitudes were standardized to the membrane potential of –70 mV (to correct the changes in the driving force of the voltage shifts upon RMP changes from one muscle fiber to another) using the formula: A_st _= A∙(−70/RMP), where A is the recorded amplitude of postsynaptic potential (MEPP or EPP) and A_st_ is the standardized amplitude of MEPP.

The MEPP and EPP amplitudes were standardized to the membrane potential of –50 mV when evoked synaptic activity was studied using cut muscle fibers (Flink & Atchison, 2003). Quantal content of EPP was calculated as a ratio between the mean standardized EPP amplitude corrected to nonlinear summation (McLachlan & Martin, 1981) and the mean standardized MEPP amplitude.

Data are presented as the mean ± *SEM*;* n* reflects number of the synapses analyzed. Statistical significance between sample means was assessed using Student's *t*‐test (in the case of normal distribution), one‐way analysis of variance ANOVA, or Mann‐Whitney test (when cumulative probabilities of MEPP amplitudes were analyzed). The difference was considered significant at *p* < 0.05.

### 2.4 Chemicals

All drugs were diluted to a final concentration in Liley solution. CGRP (rat peptide isoform which shares the same amino acid sequence with endogenous mouse peptide) and inhibitor of CGRP‐receptors—rat CGRP_8‐37_—were obtained from Bachem (Bubendorf, Switzerland). Ryanodine (an activator of RyR) and an inhibitor of protein kinase A H‐89 were obtained from Enzo Life Sciences (Farmingdale, NY, USA). Inhibitor of vesicular ACh transporter—(±) vesamicol hydrochloride, inhibitors of CaMKII—KN‐62, KN‐93, and negative control for KN‐93—KN‐92—were all from Tocris Bioscience (Ellisville, MO, USA). Stock solutions of all drugs except H‐89 and inhibitors of CaMKII were prepared in deionized water, H‐89, KN‐62, KN‐93, and KN‐92 were dissolved in dimethylsulfoxide (DMSO) (Helicon, Moscow, Russia). The final DMSO concentration did not exceed 0.01% (v/v), and at this concentration, DMSO did not affect the parameters of spontaneous ACh release in mouse motor synapses. As KN‐62, KN‐93, and KN‐92 are reported to be light sensitive, efforts were made to minimize the exposure to light during the experiments involving these drugs.

## 3 RESULTS

**Figure 1:**
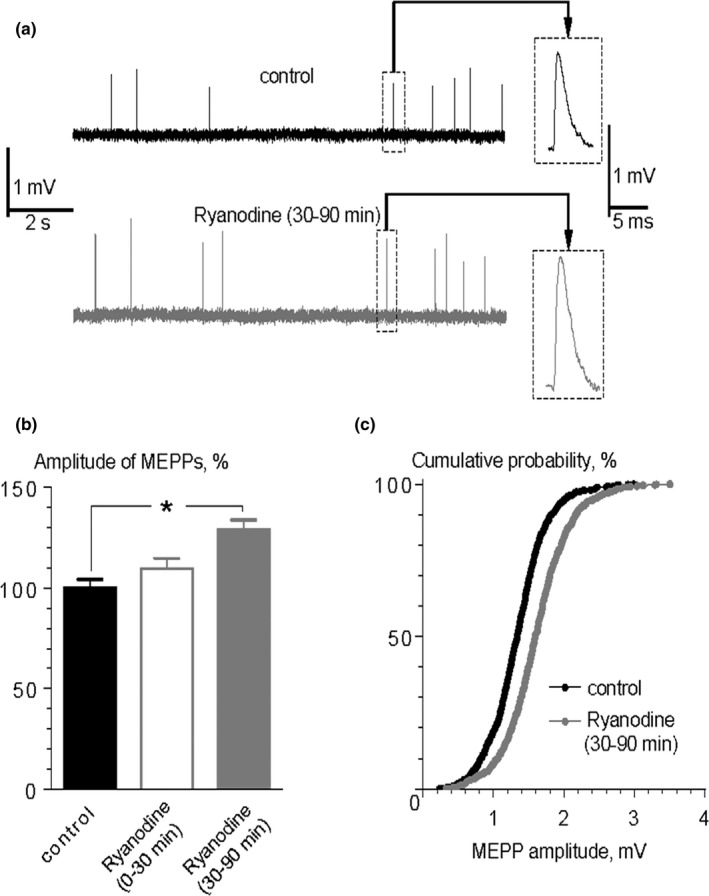
An increase in MEPP amplitude in the presence of ryanodine. (a) Representative intracellular recordings of MEPPs in control and in the presence of ryanodine (0.1 μM). Insets show the enlarged views of MEPPs. (b) Mean amplitude of MEPPs in control (*n* = 23), during first 30 min of ryanodine application (*n* = 15) and within 30–90 min of ryanodine application (*n* = 28). *p < 0.05. Histograms and error bars indicate the mean and *SEM*, respectively (c) Cumulative probability plots of MEPP amplitudes in control and in the presence of ryanodine for 30–90 min, *p* < 0.001

In the first series of experiments, we used the prolonged stimulation (for 90 min) of calcium release from intraterminal stores using application of ryanodine at submicromolar concentration (0.1 μM) as RyR activator (Balezina, 2002; Gerasimova et al., 2015; Zucchi & Ronca‐Testoni, 1997). Ryanodine did not change MEPPs amplitude during first 30 min; however, within the next 60 min of its application, MEPP amplitude increased by 29% from 1.36 ± 0.06 mV under control conditions to 1.75 ± 0.06 mV (*p* < 0.05) (Figure 1). The cumulative probability curve of MEPP amplitudes was clearly right shifted in the presence of 0.1 μM ryanodine (30–90 min) indicating that activation of RyRs induced a significant increase in MEPP amplitudes across the entire population of MEPPs (Figure 1c). Mean RMP value measured in the control was −62.73 ± 0.45 mV (*n* = 23), and within 30–90 min of ryanodine application, it was −62.41 ± 0.40 mV (*n* = 28, *p* > 0.05). No significant changes were observed in the frequency of MEPPs in the presence of 0.1 μM ryanodine (0.58 ± 0.06 Hz) in comparison to control (0.57 ± 0.05 Hz, *p* > 0.05). The time parameters of MEPPs did not change either: the rise time (10%–90%) of MEPPs was 0.86 ± 0.03 ms in control and 0.83 ± 0.03 ms (*p* > 0.05) under ryanodine; half‐decay, 2.29 ± 0.07 ms and 2.40 ± 0.07 ms in control and under ryanodine, respectively (*p* > 0.05).

**Figure 2:**
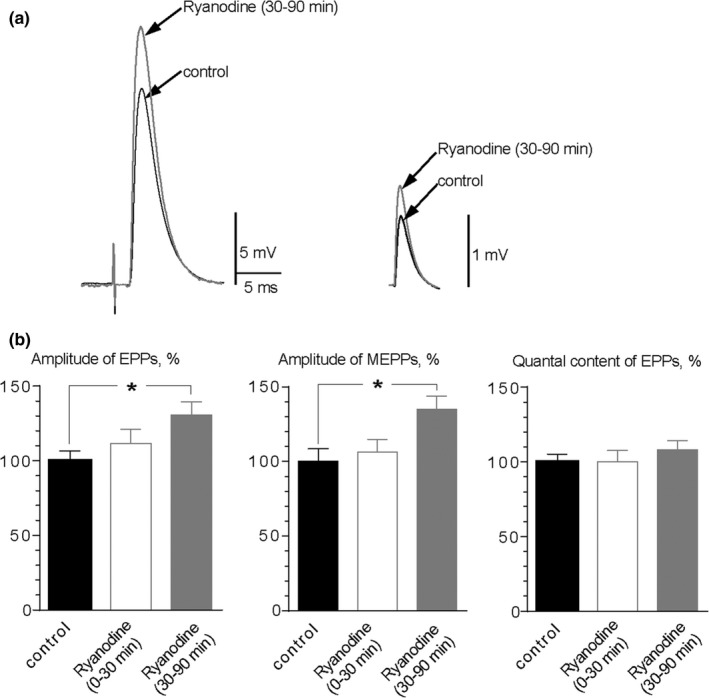
Ryanodine effect on single evoked EPPs and MEPPs. (a) Representative recordings of EPPs (left) and MEPPs (right) in control and in the presence of ryanodine for 30–90 min. (b) Mean EPPs amplitude, MEPPs amplitude, and quantal content of EPPs (left to right) in control (*n* = 40), during first 30 min of ryanodine application (*n* = 18) and within 30–90 min of ryanodine application (*n* = 27). *p < 0.05. Histograms and error bars indicate the mean and *SEM*, respectively

Next, we tested the effect of 0.1 μM ryanodine on evoked quantal ACh release. Both EPPs and MEPPs were recorded during evoked synaptic activity (stimulation frequency – 0.3 Hz). Ryanodine did not affect the RMP of muscle fibers: −33.98 ± 1.05 mV (*n* = 40) in control, −31.13 ± 1.03 mV (*n* = 18, *p* > 0.05) during first 30 min after ryanodine was applied and −31.60 ± 0.95 mV (*n* = 27, *p* > 0.05) within 30–90 min of ryanodine application. We did not reveal any changes in time parameters of EPPs or MEPPs in the presence of ryanodine. The amplitude of single evoked EPPs in control was 25.87 ± 2.18 mV and did not change significantly during first 30 min after beginning of ryanodine application (27.45 ± 2.29 mV, *p* > 0.05) but increased to 34.94 ± 2.28 mV after incubation with ryanodine for 30–90 min (*p* < 0.05) (Figure 2). The amplitude of MEPPs changed in the same way: It slightly increased from 1.08 ± 0.07 mV in control to 1.20 ± 0.10 mV (*p* > 0.05) during first 30 min of ryanodine presence in bath solution followed by a significant increase by 30% to 1.41 ± 0.10 mV within 30–90 min after application of ryanodine (*p* < 0.05). EPPs quantal content did not change under ryanodine (control—24.09 ± 1.24; 0–30 min after ryanodine application—24.14 ± 1.81, 30–90 min after ryanodine application 26.00 ± 1.55 mV, *p* > 0.05) (Figure 2).

**Figure 3:**
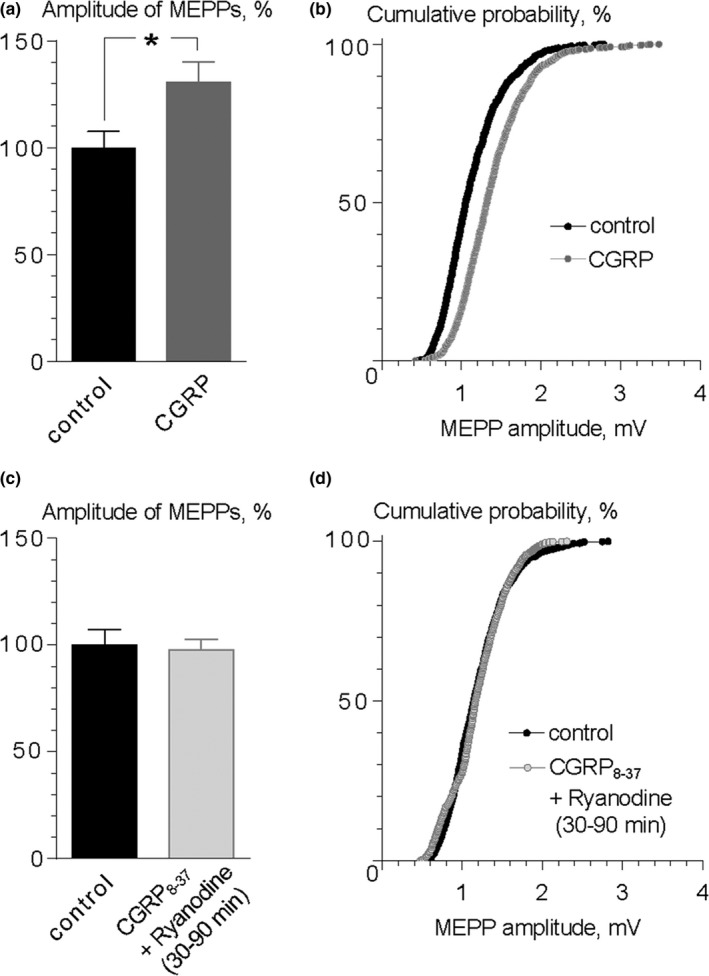
CGRP mediates the ryanodine‐induced increase in MEPP amplitude. (a) Mean amplitude of MEPPs in control (*n* = 16) and during application of exogenous CGRP (10 nM) for 40 min (*n* = 18). *p < 0.05. Histograms and error bars indicate the mean and *SEM*, respectively. (b) In the same way as for ryanodine, CGRP shifted the curve of MEPP amplitude cumulative probability to the right, *p* < 0.001. (c) Preapplication of CGRP_8‐37_ (1 μM) prevented the effect of ryanodine (0.1 μM). Histograms and error bars indicate the mean and *SEM*, respectively. (d) Blocking of CGRP receptors with CGRP_8‐37_ prevents the rightward shift of cumulative probability plot of MEPP amplitudes in the presence of ryanodine, *p* = 0.57

This potentiating effect of ryanodine definitely resembled the action of exogenous CGRP on MEPPs and EPPs amplitudes that we recently described in mouse diaphragm synapses (Gaydukov et al., 2016). Similar to ryanodine, application of exogenous CGRP (10 nM) induced an increase in the MEPPs amplitude of the MEPPs up to 130.6 ± 9.6% (*p* < 0.05) (Figure 3a) without changing muscle fibers RMP, and the time parameters or frequency of MEPPs. The cumulative probability plots revealed a shift toward the larger amplitudes under CGRP, as in the case of ryanodine (Figure 3b). On the other hand, the effect of the exogenous peptide required less time to develop (within 30–40 min of application) than the analogous effect of ryanodine.

In our previous report, we have demonstrated that potentiating action of ryanodine on MEPPs amplitude was prevented by loading the nerve terminal with Ca^2+^ chelator EGTA‐AM suggesting that an increase in MEPPs amplitude under ryanodine is calcium dependent (Skiteva et al., 2012). We hypothesized that the release of stored calcium by ryanodine may selectively stimulate the exocytosis of CGRP‐containing LDCVs, which will be followed by the increase in MEPP amplitude as the result of activation of CGRP receptors by released endogenous CGRP. To test this hypothesis, we studied the action of 0.1 μM of ryanodine in the presence of truncated CGRP (CGRP_8‐37_, 1 μM) which is widely used as CGRP‐receptor blocker (Gaydukov et al., 2016; Han, Adwanikar, Li, Ji, & Neugebauer, 2010; Hay, Garelja, Poyner, & Walker, 2017; Wu et al., 2015). We have shown that CGRP_8‐37_ alone had no significant effect on MEPP amplitude (Gaydukov et al., 2016). Nevertheless, when CGRP‐receptors were blocked, application of ryanodine (0.1 μM) did not significantly change the amplitude of MEPPs (Figure 3c,d). These data suggest that ryanodine initiates calcium‐dependent release of endogenous CGRP, which, in turn, potentiates MEPP amplitude. If it is the case, the mechanism that underlies the increase in MEPP amplitude by endogenous CGRP must be the same as for the exogenously applied peptide. As we have shown recently, this mechanism includes the presynaptic action of CGRP which stimulates ACh transport into synaptic vesicles and increases ACh quantal size (Gaydukov et al., 2016).

**Figure 4:**
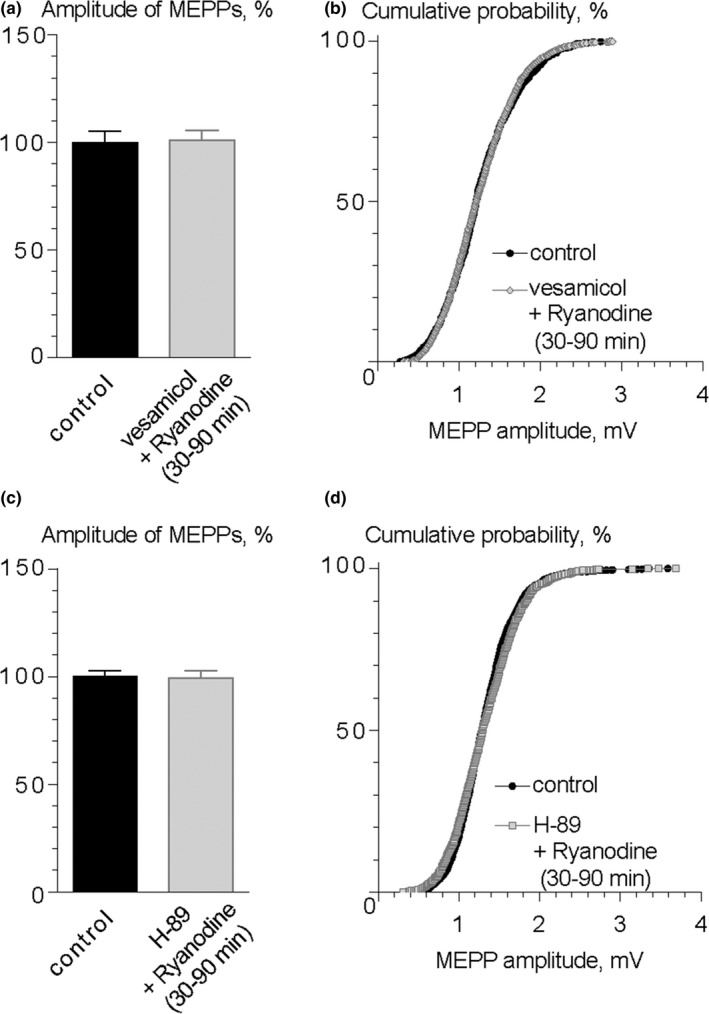
Intracellular mechanisms of ryanodine action at the mouse NMJs. (a) Vesicular ACh transporter blocker vesamicol (1 μM) prevented MEPP amplitude increase induced by ryanodine (0.1 μM). Histograms and error bars indicate the mean and *SEM*, respectively. (b) The overlapping of cumulative probability plots of MEPP amplitudes in control and during application of ryanodine in the presence of vesamicol, *p* = 0.78. (c) Inhibition of PKA by H‐89 (0.1 μM) prevents the ryanodine‐induced increase in MEPP amplitude. Histograms and error bars indicate the mean and *SEM*, respectively. (d) Cumulative probability plots of MEPP amplitudes in control and during application of ryanodine in the presence of H‐89, *p* = 0.60

Taking into account these arguments, we next examined whether vesamicol (direct inhibitor of vesicular ACh transporter) may counteract the effect of ryanodine. In our previous studies, we have shown that vesamicol by itself has no significant effect on MEPP amplitude (Gaydukov & Balezina, 2006; Gaydukov et al., 2016). However, application of ryanodine (0.1 μM) in the presence of vesamicol (1 μM) did not significantly alter the MEPP amplitude: 1.25 ± 0.07 mV (*n* = 22) in control and 1.28 ± 0.06 mV (*n* = 26, *p* > 0.05) under ryanodine (30–90 min) with vesamicol (Figure 4a). No shift in cumulative probability curve of MEPPs amplitude was observed (Figure 4b). Hence, the blocking of vesicular transport of ACh completely prevented the ryanodine‐induced increase in MEPP amplitude.

It is known that an increase in the amplitude of uniquantal MEPPs due to quantal size upregulation in amphibian and mammalian motor synapses under various stimuli, including exogenous CGRP, requires the activity of PKA (Gaydukov et al., 2016; Van der Kloot, Benjamin, & Balezina, 1998; Van der Kloot & Brănişteanu, 1992). To explore the role of PKA in the ryanodine‐induced increase in MEPP amplitude, we tested the effect of ryanodine in the presence of PKA inhibitor H‐89 (1 μM). We found that 0.1 μM ryanodine was not able to increase the MEPP amplitude when PKA is inhibited (Figure 4c,d). Altogether, these results suggest that the stimulation of stored calcium release in mouse motor synapses may provoke exocytosis of endogenous CGRP which, like exogenous CGRP, increases MEPP amplitude via quantal size upregulation.

**Figure 5:**
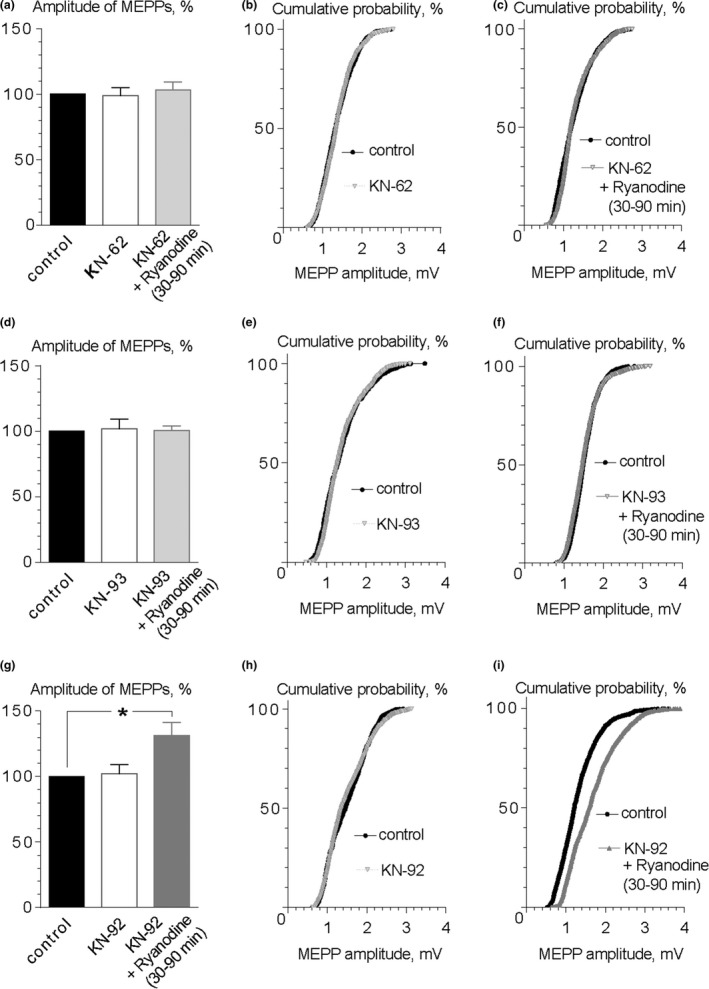
The potentiating effect of ryanodine on the amplitude of MEPPs is mediated by CaMKII. (a) Mean amplitude of MEPPs in control (*n* = 15 when only KN‐62 was applied subsequently and *n* = 16 when ryanodine was applied in the presence of KN‐62), during application of 3 μM KN‐62 (*n* = 18) and ryanodine (0.1 μM) in the presence of KN‐62 (*n* = 26). Histograms and error bars indicate the mean and *SEM*, respectively. (b) Cumulative probability plots of MEPP amplitudes in control and in the presence of KN‐62, *p* = 0.42. (c) Cumulative probability plots of MEPP amplitudes in control and during application of ryanodine in the presence of KN‐62, *p* = 0.11. (d) Mean amplitude of MEPPs in control (*n* = 16 when only KN‐93 was applied subsequently and *n* = 17 when ryanodine was applied in the presence of KN‐93), during application of 3 μM KN‐93 (*n* = 21) and ryanodine (0.1 μM) in the presence of KN‐93 (*n* = 31). Histograms and error bars indicate the mean and *SEM*, respectively. (e) Cumulative probability plots of MEPP amplitudes in control and in the presence of KN‐93, *p* = 0.12. (f) Cumulative probability plots of MEPP amplitudes in control and during application of ryanodine in the presence of KN‐93, *p* = 0.14. (g) Mean amplitude of MEPPs in control (*n* = 19 when only KN‐92 was applied subsequently and *n* = 21 when ryanodine was applied in the presence of KN‐92), during application of 3 μM KN‐92 (*n* = 26) and ryanodine (0.1 μM) in the presence of KN‐92 (*n* = 26). Histograms and error bars indicate the mean and *SEM*, respectively. (e) Cumulative probability plots of MEPP amplitudes in control and in the presence of KN‐92, *p* = 0.58. (f) Cumulative probability plots of MEPP amplitudes in control and during application of ryanodine in the presence of KN‐92, *p* < 0.001

One question remained unresolved—how the endogenous CGRP exocytosis is mediated after calcium release from presynaptic ryanodine‐sensitive calcium stores? At *Drosophila* NMJs, it was suggested that calcium release from presynaptic endoplasmic reticulum activates CaMKII which, in turn, facilitates the mobilization and secretion of LDCVs (Shakiryanova et al., 2007). We investigated whether this protein kinase in mouse motor synapses can mediate the effect of ryanodine and participate in the release of endogenous CGRP which increases MEPP amplitude. We tested the effects of KN‐62 and KN‐93, inhibitors of CaMKII, as well as KN‐92, an inactive analogue of KN‐93 during ryanodine application. We found that none of these drugs affected MEPP amplitude during their application for 90 min (i.e. during the time span we used for testing the ryanodine effect) (Figure 5a,b,d,e). The ryanodine‐induced increase in MEPP amplitude was not observed in the presence of both CaMKII inhibitors (Figure 5a,c,d,f). Moreover, KN‐92, which was used as the negative control for KN‐93, was unable to prevent the increase in MEPP amplitude by 30% after ryanodine application: 1.34 ± 0.11 mV (*n* = 21) in control and 1.77 ± 0.13 mV (*n* = 26, *p* < 0.05) under ryanodine with KN‐92 (Figure 5g,i). These results suggest that, in mouse NMJs, the activation of CaMKII by calcium from ryanodine‐sensitive calcium stores is necessary for initiation of endogenous CGRP exocytosis which is followed by the increase in ACh quantal size.

**Figure 6:**
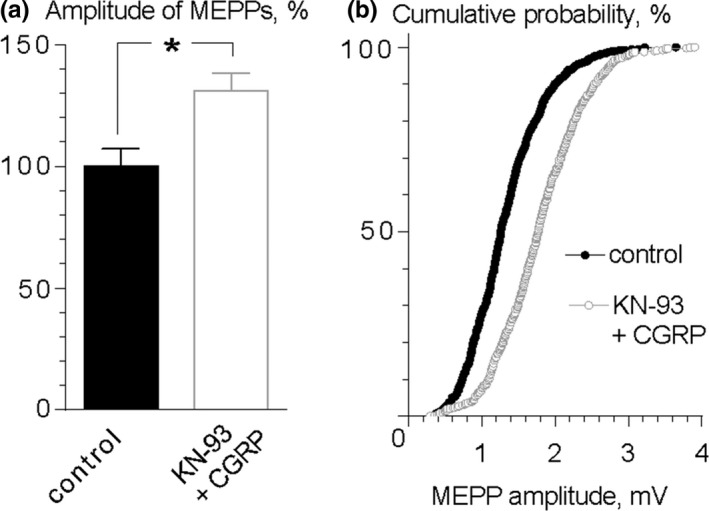
CaMKII does not act as downstream effector after activation of CGRP receptors. (a) Mean amplitude of MEPPs in control (*n* = 15) and during application of CGRP (10 nM) in the presence of KN‐93 (*n* = 19). *p < 0.05. Histograms and error bars indicate the mean and *SEM*, respectively. (b) Cumulative probability plots of MEPP amplitudes in control and during application of CGRP in the presence of KN‐93. *p* < 0.001

Finally, to rule out the possibility of CaMKII participation in signaling mechanism downstream activation of CGRP receptors leading to an increase in MEPP amplitude, we tested the effect of exogenous CGRP (10 nM) in the presence of CaMKII inhibitor KN‐93 (3 μM). Unlike blocking effect of KN‐93 on ryanodine‐induced MEPP enlargement, inhibition of CaMKII was not able to prevent the potentiating effect of exogenous CGRP on the amplitudes of MEPPs: the mean MEPP amplitude was 1.33 ± 0.10 mV (*n* = 15) in control and 1.74 ± 0.09 mV (*n* = 19, *p* < 0.05) in the presence of CGRP and KN‐93 (Figure 6). These data suggest that CGRP, once released to synaptic cleft, does not utilize CaMKII as its downstream effector after activation of CGRP receptors.

## 4 DISCUSSION

The present study revealed that ryanodine action in motor synapses of mice can initiate an increase in postsynaptic potentials amplitude due to presynaptic action of endogenous CGRP. The ryanodine effect is mediated by enhanced activity of CaMKII. We assume that this protein kinase being activated by calcium release from Ca^2+^‐stores may selectively facilitate exocytosis of LDCVs and endogenous CGRP release into synaptic cleft. We found that the ryanodine‐induced enlargement of MEPP amplitude may be prevented by blockage of CGRP‐receptors, by direct inhibition of vesicular transport of ACh or by inhibition of PKA activity. This suggests that endogenous CGRP, when it is released and accumulated in the synaptic cleft, may act on its presynaptic receptor and trigger the PKA‐dependent enhanced ACh transport into synaptic vesicles.

At present, the mechanisms leading to effective and selective release of endogenous CGRP in motor synapses and the spectrum of its acute effects remain poorly understood. Previously, some attempts were made to evoke the exocytosis of endogenous CGRP in various NMJs using high external K^+^ concentration (Sakaguchi et al., 1991; Van der Kloot et al., 1998) or high‐frequency nerve stimulation (Kimura et al., 1997; Sala et al., 1995). It has been shown that endogenous peptide after its release could change membrane or metabolic characteristics of muscle fibers (Macdonald, Nielsen, & Clausen, 2008; Sala et al., 1995; Uchida et al., 1990). In our study, another procedure was used to stimulate selectively the release of endogenous CGRP—prolonged stimulation of stored calcium release by ryanodine as agonist of RyRs. It is well‐known that ryanodine at submicromolar concentrations locks the RyRs in open configuration but with reduced conductivity and thus promoting leakage of calcium from the stores (Clarke & Hendrickson, 2016; Nagasaki & Fleischer, 1988). It has been shown that ryanodine‐induced Ca^2+^‐release from Ca^2+^‐stores is insufficient to increase the frequency of MEPPs. Ryanodine activation of RyRs may affect the frequency of MEPPs (the rate of standard cholinergic vesicles exocytosis) only when additional preliminary depolarization and Ca^2+^‐influx via voltage‐dependent calcium channels of nerve terminal is applied (Balezina, Ermishina, & Lapteva, 2004; Nishimura, Tsubaki, Yagasaki, & Ito, 1990). Here, we found that the ryanodine application to resting NMJs of mice is sufficient for potentiation of MEPPs amplitude mediated by endogenous CGRP action, as ryanodine effect may be prevented by blockage of CGRP receptors. We suggest that ryanodine can induce calcium release from Ca^2+^‐stores, which appears to be associated with both localization of the LDCVs in the nerve terminal and the Ca^2+^‐dependent mechanism of their trafficking and exocytosis (van de Bospoort et al., 2012; Gondré‐Lewis, Park, & Loh, 2012; Nurrish, 2014). Our suggestion is supported by previous results describing that the pool of the LDCVs is localized away from the extracellular calcium influx at the active zones (Pécot‐Dechavassine & Brouard, 1997), as well as ryanodine‐sensitive Ca^2+^‐stores (Bouchard, Pattarini, & Geiger, 2003). In addition, the RyR‐mediated LDCV mobilization, trafficking, and exocytosis have been recently described at Drosophila NMJs (Shakiryanova, Zettel, Gu, Hewes, & Levitan, 2011; Shakiryanova et al., 2007; Wong, Shakiryanova, & Levitan, 2009).

We found that the ryanodine‐induced enlargement of MEPPs amplitude may be prevented not only by blockage of CGRP receptors, but also by direct inhibition of vesicular transport of ACh by vesamicol, and by inhibition of PKA activity. The same procedures have been found to prevent the potentiating effect of exogenous CGRP on MEPPs amplitude (Gaydukov et al., 2016). Thus, we revealed common pathways and signaling mechanisms for both ryanodine‐induced increase in MEPPs amplitude and enlargement of MEPPs amplitude by exogenous CGRP.

However, we also observed a significant difference in dynamics of exogenous CGRP effects (30–40 min) and ryanodine‐induced effects (mediated by endogenous CGRP). In the latter case, about 1.5 hr from the start of ryanodine application is required for the detection of a significant increase in MEPPs amplitude. There may be a number of several reasons for this discrepancy. First, the LDCV exocytosis proceeds more slowly than the exocytosis of small synaptic vesicles with conventional neurotransmitter (Wong, Cavolo, & Levitan, 2015; Xia, Lessmann, & Martin, 2009). Secondly, as we have shown, the release (leakage) of calcium from calcium stores with the subsequent activation of CaMKII is required to trigger the exocytosis of LDCVs, which in total also requires a certain time. Finally, it has been shown that the size of LDCV pool in motor nerve terminals is relatively small (Kashihara, Sakaguchi, & Kuno, 1989; Pécot‐Dechavassine & Brouard, 1997). So it takes time to increase the concentration of endogenous CGRP in synaptic cleft to the level sufficient for activation of CGRP receptors. All these time‐consuming stages are absent during the direct application of exogenous CGRP, whose effect on the MEPP amplitude develops more rapidly.

The analysis of ryanodine effect on MEPPs amplitude in the presence of CaMKII blockers provided evidence of involvement of this protein kinase in the release of endogenous CGRP. Over the last years, the crucial role of CaMKII in the LDCV maturation, their axonal transport, and especially mobilization of LDCV to their release sites in nerve terminals are actively discussed (Hoover et al., 2014; Nurrish, 2014). The results of our work suggest that triggering of CGRP‐containing LDCVs exocytosis requires not only the mandatory release of calcium from the ryanodine‐sensitive Ca^2+^‐stores but also the subsequent activation of CaMKII. Taking into account the data obtained at *Drosophila* NMJs (Shakiryanova et al., 2007, 2011), our results suggest that conservative signaling pathway exists in NMJs of different species, which regulates the exocytosis of LDCVs via RyRs and CaMKII. Interestingly, the increase in ACh quantal size during exogenous CGRP application could not be prevented by CaMKII blocking. These data indicate that CaMKII participation in the quantal size increase is caused by the activation of this protein kinase after release of stored calcium, and this occurs before exocytosis of endogenous CGRP and its subsequent activation of CGRP receptors.

Our hypothesis of presynaptic RyRs stimulation followed by presynaptic CaMKII activation does not exclude the ability of ryanodine to initiate RyR‐mediated calcium release and CaMKII activation in muscle fibers and/or perisynaptic Schwann cells of NMJs. However, activation of postsynaptic or glial RyRs and CaMKII cannot provide endogenous CGRP release and subsequent CGRP‐mediated increase in MEPPs amplitude, as muscle fibers and Schwann cells both lack significant CGRP expression and release. It has been reported that CGRP is neurogenic and is released from motor nerve terminals in different tissues (Uchida et al., 1990; Sakaguchi et al., 1991; Macdonald et al., 2008; Iyengar et al. 2017). Thus, it looks more likely that ryanodine application to motor synapses initiates calcium release from presynaptic Ca^2+^‐stores leading to secretion of endogenous CGRP from motor nerve terminals followed by an increase in MEPPs amplitude in motor synapses.

In addition to the well‐known neurotrophic effects of CGRP aimed to maintain the properties of skeletal muscle fibers, a new form of endogenous CGRP activity in motor synapses is revealed. We found for the first time that endogenous CGRP, which is released in the synaptic cleft due to the activation of ryanodine sensitive Ca^2+^‐stores, can significantly increase the amplitudes of MEPPs and EPPs. This acute action of endogenous CGRP may considerably potentiate the synaptic transmission as an increase in MEPPs amplitude also affects the amplitude of the multiquantal EPPs. This means that CGRP may be able to amplify the synaptic transmission mediating a positive feedback loop raising ACh quantal size. This mechanism of synaptic plasticity (increase in quantal size), poorly explored either at the central or at the peripheral synapses, may serve for improving the safety factor of neuromuscular transmission, for example, during prolonged synaptic activity, especially at high frequencies of stimulation.

In summary, our data allow to consider the new mechanism of ryanodine‐induced increase in postsynaptic endplate potentials amplitude at mouse NMJs. Although being indirect, the obtained results in conjunction with our previously reported and literature data allow to propose the hypothesis that ryanodine‐ and RyR‐dependent calcium release from the Ca^2+^‐stores in nerve terminals is followed by activation of presynaptic CaMKII leading to endogenous CGRP release from mouse motor nerve terminals. The effect of endogenous CGRP is presynaptic and mediated by CGRP‐receptors and PKA activation which stimulates filling of synaptic vesicles with ACh and an increase in ACh quantal size.

Identification of the presynaptic CaMKII particular targets in release machinery of CGRP‐containing LDCVs and the evaluation of normal or pathological conditions of synaptic activity when this calcium‐ and CGRP‐dependent mechanism may be triggered requires further research.

## CONFLICT OF INTEREST

The authors state no conflict of interest.
